# Evaluation of Analysis Methods for Formaldehyde, Acetaldehyde, and Furfural from Fast Pyrolysis Bio-oil

**DOI:** 10.1021/acs.energyfuels.1c02208

**Published:** 2021-10-29

**Authors:** Taina Ohra-aho, Léon Rohrbach, Jozef G. M. Winkelman, Hero J. Heeres, Atte Mikkelson, Anja Oasmaa, Bert van de Beld, Evert. J. Leijenhorst, Hans Heeres

**Affiliations:** † VTT Technical Research Centre of Finland Ltd., P.O. Box 1000, FI-02044 Espoo, Finland; ‡ Department of Chemical Engineering (ENTEG), University of Groningen, Nijenborgh 4, 9747 AG Groningen, The Netherlands; § BTG Biomass Technology Group BV, P.O. Box 835, 7500 AV Enschede, The Netherlands

## Abstract

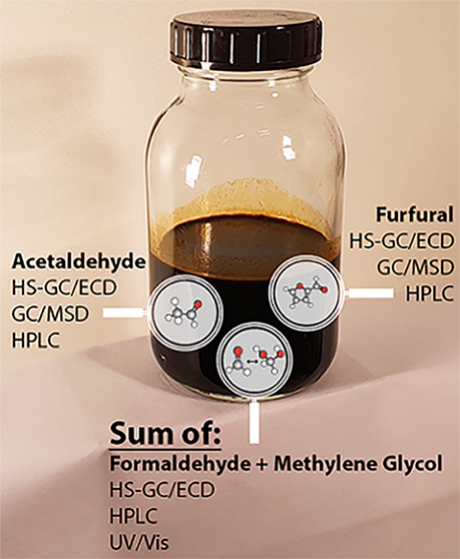

Fast pyrolysis bio-oil (FPBO), a second-generation liquid bioenergy carrier, is currently entering the market. FPBO is produced from biomass through the fast pyrolysis process and contains a large number of constituents, of which a significant part is still unknown. Various analytical methods have been systematically developed and validated for FPBO in the past; however, reliable methods for characterization of acetaldehyde, formaldehyde, and furfural are still lacking. In this work, different analysis methods with (HS-GC/ECD, HPLC, UV/Vis) and without derivatization (GC/MSD, HPLC) for the characterization of these components were evaluated. Five FPBO samples were used, covering a range of biomass materials (pine wood, miscanthus, and bark), storage conditions (freezer and room temperature), and after treatments (none, filtration, and vacuum evaporation). There was no difference among the methods for the acetaldehyde analysis. A significant difference among the methods for the determination of formaldehyde and furfural was observed. Thus, more data on the accuracy of the methods are required. The precision of all methods was below 10% with the exception of the HPLC analysis of acetaldehyde with an RSD of 14%. The concentration of acetaldehyde in the FPBO produced from the three different biomasses and stored in a freezer after production ranged from 0.24 to 0.60 wt %. Storage at room temperature and vacuum evaporation both decreased significantly the acetaldehyde concentration. Furfural concentrations ranged from 0.11 to 0.36 wt % for the five samples. Storage and after treatment affected the furfural concentration but to a lesser extent than for acetaldehyde. Storage at room temperature decreased formaldehyde similarly to acetaldehyde; however, after vacuum-evaporation the concentration of formaldehyde did not change. Thus, the analysis results indicated that in FPBO the equilibrium of formaldehyde and methylene glycol is almost completely on the methylene glycol side, as in aqueous solutions. All three methods employed here actually measure the sum of free formaldehyde and methylene glycol (FAMG).

## 1 Introduction

Fast pyrolysis is at its early stage of commercialization with demonstration plants in Finland, The Netherlands, the United States, and Canada and new plants under construction or design in Canada, Finland, and Sweden. Based on these plants the total fast pyrolysis bio-oil (FPBO) production capacity has been estimated to exceed 180 000 tonnes in 2021. The FPBO is commercially used in boilers for heat and energy and in turbines for process steam. ASTM and EN standards exist for FPBO use in industrial boilers, and a technical report has been prepared for IC-engine use. For market introduction both standards and Registration, Evaluation and Authorization of Chemicals (REACH) registration are needed.^1^ In the European Union (EU), the chemical regulation system REACH has been introduced, which means that a registration with the European Chemical Agency (ECHA) must be made if FPBO is produced in or imported to the EU.^2^ FPBO cannot be sufficiently identified by its chemical composition, because the number of constituents is large and the composition is, in a significant part, unknown (a UVCB, Substances of Unknown or Variable Composition, Complex reaction products, or Biological Materials).^3^ Therefore, the main identifiers of FPBO are the source and the process used. Some additional properties and compositions of FPBO have been defined for REACH (Table 1) and limit values have been set.^2^ Analytical methods have been systematically developed and validated, and some of the methods have already been standardized. However, reliable methods for characterization of polar components including formaldehyde, acetaldehyde, and furfural from FPBO are still lacking. Monitoring of these compounds is important, due to the potential health effect to human.

**Table 1:** Properties and Composition of FPBO for REACH^2^

polar components	value	parameter	value
formaldehyde	<0.5 wt %	pH	>2–3.5
methanol	<3 wt %	water content	<40 wt %
		ash content	<0.5 wt %
nonpolar components		solids content	<5.0 wt %
PAH13^a^	<35 mg/L	viscosity (40 °C)	<200 mm^2^/s
benzo[*a*]pyrene	<0.01 wt %	density	1.1–1.3 kg/dm^3^
dibenz[*a*,*h*]anthracene	<0.01 wt %		
sum of Carc. 1B classified substances^b^	<0.1 wt %		
sum of Carc. 2 classified substances^c^	<1.0 wt %		

a Sum PAH13: anthracene, benz[*a*]anthracene, benzo[*a*]pyrene, benzo[*a*]fluoranthene, benzo[*k*]fluoranthene, benzoperylene, chrysene, dibenz[*a*,*h*]anthracene, fluorene, fluoranthene, indenopyrene, phenantrene, pyrene.

b Carc. 1B classified substances (Annex VI of CLP regulation 1272/2008), e.g., of sum PAH13: benz[*a*]anthracene, benzo[*a*]pyrene, benzo[*k*]fluoranthene, chrysene, dibenz[*a*,*h*]anthracene.

c Carc. 2 classified substances (Annex VI of CLP regulation 1272/2008): e.g., formaldehyde, acetaldehyde, furfural.

Different chromatographic techniques with and without derivatization have been used for aldehydes analysis from FPBO. The sensitivity of the analysis improves and the matrix effect can be minimized applying aldehydes selective derivatization agents. One of the most common reagents used is *o*-(2,3,4,5,6-pentafluorobenzyl) hydroxylamine hydrochloride (PFBHA) reagent that forms aldehyde selective oxime-derivatives.^4^ For the quantitative analysis of the volatile oximes, a headspace (HS) combined with gas chromatography and a mass selective detector (GC/MSD) has been applied.^5^ The second approach has been a solid phase microextraction (SPME) with direct on-fiber derivatization of aldehydes. A fiber coated with the PFBHA phase forms oximes with aldehydes simultaneously during extraction. After extraction, formed oximes are released in a GC injector for the GC/MSD analysis.^5^ Third, ketones and aldehydes have been converted into hydrazones by using 2,4-dinitrophenylhydrazine (DNPH) derivatization.^6^ The separation and quantitative determination of DNPH derivatives has been performed by HPLC-UV. However, it has been reported that the method is not suitable for the quantification of aldehydes from FPBO due to the side reactions of reagents and FPBO components.^5^ For formaldehyde analysis, UV/Vis spectrometry at 412 nm after formation of the complex diacetyldihydrolutidine by using acetylacetone and ammonium acetate has been applied.^7,8^ The formed complex absorbs light at 412 nm and can be used to quantify formaldehyde from various types of samples selectively. The method has not earlier been used to determine formaldehyde in FPBO. Therefore, in this study it will be applied for FPBOs and compared with other available methods. Headspace^9,10^ and full evaporation technique (FET) headspace^10^ followed by GC-FID/MSD analysis without derivatization has been used for the volatile’s determination including formaldehyde and acetaldehyde from FPBO. In both methods, the sample is kept for a selected time at elevated temperature. After the equilibrium between the liquid and the gas phase is reached, vapor sampling from the headspace is performed and subsequently injected in the GC-FID/MSD for the analysis. In the HS method sodium chloride (NaCl) solution can be added to the sample to improve the polar volatile compounds release from the liquid to gas phase.^10^ In the FET method only a small quantity of FPBO is added into the vial, ensuring maximum headspace for the volatiles to evaporate. Furfural has seldom been determined by HS methods. Instead, GC-FID/MSD analysis after water extraction of FPBO has been favored.^9^ In addition, furfural has been determined by GC after oximation with hydroxylamine hydrochloride and the following trimethylsilylation with N,O-bis(trimethylsilyl)trifluoroacetamide (BSTFA).^11^

Other thing to be addressed is that aldehydes, especially formaldehyde in polar solvents such as water, alcohols, and acids, can polymerize and/or react with the solvent. More specifically, in water formaldehyde hydrates rapidly to form methylene glycol. Practically, standard analytical methods cannot distinguish between both components, but chemically and toxicologically both components are certainly not equivalent.^12^ The equilibrium between formaldehyde and methylene glycol is described by eq 1:
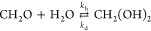
1

Winkelman et al.^13^ established a correlation for the chemical equilibrium constant of the hydration of formaldehyde in water, *K*_h_, from the ratio of the hydration and dehydration rate constants, *k*_h_ and *k*_d_, that were determined independently using two different methods (eq 2). The rate constant of formaldehyde hydration was obtained from the chemically enhanced absorption of formaldehyde into water in a stirred tank reactor at temperatures between 20 and 65 °C.^13^ The dehydration rate of methylene glycol was obtained indirectly by trapping the formaldehyde formed using sulphite as a trapping agent or chemical scavenger.^14^
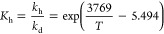
2

**Figure 1:**
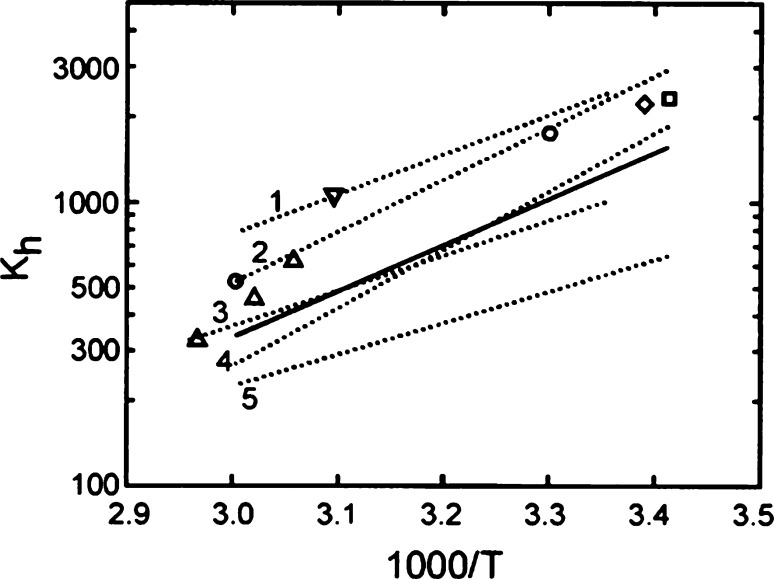
Equilibrium constants for FA-MG equilibrium. Solid line: Winkelman et al.^13^ Dotted lines and symbols: other literature data; references can be found in ref (13). Reprinted with permission from from Winkelman et al.^13^ Copyright 2002 Elsevier.

The results obtained by Winkelman et al.^13^ are shown in Figure 1 (solid-line) and fall well within the data of other researchers. Experimental data of several researchers is also given in Figure 1. (dotted lines and markers), and generally the values are somewhat higher than those calculated by the expression of Winkelman.^13^

More recently, Rivlin et al.^15^ applied ^1^H and ^13^C NMR to determine the equilibrium constant of formaldehyde hydration and dimerization in D_2_O solutions at various pH levels (2.1–7.4) and temperatures (19–63 °C). The values of the hydration equilibrium constants obtained by Rivlin et al.,^15^ as well as their temperature dependencies, are comparable to the data obtained by Winkelman et al.^13^ In all cases the hydrated form (i.e., methylene glycol) is strongly preferred, and it is concluded that in aqueous media and for temperatures between 5 and 65 °C the equilibrium between formaldehyde and methylene glycol is almost completely on the side of methylene glycol. The equilibrium constant ranges from 200 to 300 for high temperature (∼65 °C) to more than 2000 at room temperature.

This paper focuses on presenting different methods for characterization of formaldehyde, acetaldehyde, and furfural compounds. These compounds have been listed in REACH including the limit values that can exist in FPBO for registration. However, reliable methods required to determine these compounds accurately from FPBO are still missing. For comparison, the methods proposed in this paper are compared using various FPBO samples. In addition, the effect of post-treatment and aging of the FPBOs on aldehydes concentration is taken into account. In an aqueous solution, formaldehyde is in equilibrium with methylene glycol. However, analysis methods determine both these two compounds together as formaldehyde. Thus, discussion on the equilibrium of formaldehyde and methylene glycol in FPBO is provided.

## 2 Experimental Section

### 2.1 Materials

The FPBOs used in this method comparison are presented in Table 2. The samples were selected to cover a wide range of products potentially relevant for future commercial applications. Clean pine wood (stem wood) was used as “reference” material for the production of FPBO, as clean pine wood is known to yield a high quality FPBO and is often used in research and development work. The pine wood was purchased from *Bemap Houtmeel BV (Bemmel, The Netherlands).* Miscanthus is a perannial grass, which is often considered as “energy crop” in biomass cultivation systems. The miscanthus material was purchased in pelletized form from *Sieverdingbeck Miscanthus GmbH (Velen-Ramdorf, Germany)* and consisted of the miscanthus stems harvested after winter. The miscanthus pellets were dried and grinded before usage. Bark was obtained as a third biomass feedstock from *Foreco BV (Dalfsen, The Netherlands)* and is the residual product from a sawmill where softwood is debarked and peeled to generate clean wood stems. The bark was ground in a hammermill to a size below ∼5 mm and dried before use. All materials were converted to FPBO in BTG’s pyrolysis pilot plant under similar operating conditions (average pyrolysis temperature 500 °C, vapor residence time < 2 s, condenser temperature < 40 °C).

**Table 2:** FPBO Samples Used in the Evaluation of the Analysis Methods

short name^a^	feedstock	storage conditions	after treatment
pine, aged	pine	aged 1 year at room temperature	no
pine, filtrated	pine	fresh, stored in freezer (−15 °C)	filtration^b^
pine, vacuum evaporated	pine	fresh, stored in freezer (−15 °C)	vacuum evaporation^c^ + filtration^b^
miscanthus, fresh	miscanthus	fresh, stored in freezer (−15 °C)	no
bark, fresh	bark	fresh, stored in freezer (−15 °C)	no

a Samples produced in EU Residue2Heat project (GA No. 654650) by BTG.

b Pressurized filtration over a fixed mesh (10 μm).

c Vacuum evaporation at 80 °C and 100 mbar using a laboratory rotavapor; 23 wt % of FPBO was evaporated.

The possible influence of storage conditions (aged versus fresh) and after treatment was included in the sample selection as well (see Table 2). From the pine wood FPBO, one sample was aged by storing it at room temperature for 1 year, without after treatment. The second pine-wood derived sample was filtered and stored in the freezer directly after production. The third sample was treated by vacuum evaporation and filtration in order to investigate if the concentrations of formaldehyde, acetaldehyde, and furfural will be affected by vacuum evaporation of water and light organics.

### 2.2 Procedure for Shipping and Handling of the Samples

The compositions of FPBOs do change in time and are influenced by temperature.^16^ Hence, the procedure for sample shipping and handling was set up carefully. The FPBO samples produced and described in Table 2 were stored in a freezer after production, except for the aged sample. From the stored bulk samples, five subsamples of 100 g each were taken after the bulk sample was allowed to reach room temperature and after mixing to ensure homogeneous liquids. One set of subsamples was sent to VTT, one was sent to RUG, one was used by BTG, and the remaining subsamples (2 sets of 5 samples) were stored in a large freezer at BTG for possible future analysis by other laboratories. The samples were packaged in isolated containers containing frozen “cold packs” to prevent aging reactions during transport.

After arrival, the samples were stored in a freezer. For analyses, the bio-oils were taken from the freezer, allowed to reach room temperature, and homogenized by mixing for 1 h; subsequently, the samples were prepared for analysis as described in the following part.

### 2.3 Methods

#### 2.3.1 HS-GC/ECD

Static headspace–gas chromatography/electron capture detector analysis was conducted for formaldehyde, acetaldehyde, and furfural. Formaldehyde, acetaldehyde, and furfural were analyzed as oximes using an Agilent 7697A Headspace Sampler coupled with Agilent 7890B gas chromatography, and the compounds were detected using a Micro Electron Capture Detector. Due to the high sensitivity of the ECD detector, samples were diluted with water prior derivatization as follows: 10 mg of well homogenized FPBO was first diluted with 100 mL of water, and at the second stage the water-soluble fraction was diluted in a ratio of 1:50 or 1:100 depending on the compound studied and compound concentration in the sample. Formaldehyde was analyzed separately, because its concentration was higher in comparison to the acetaldehyde and furfural. First an aqueous solution of the derivatization agent O-(2,3,4,5,6-pentafluorobenzyl)-hydroxylamine (PFBOA Sigma-Aldrich) was prepared at a concentration of 6 g/L. A total of 100 μL of PFBOA solution (6 g/L) with 10 mL of diluted samples for formaldehyde and 5 mL of diluted samples for acetaldehyde and furfural analyses were placed in a 20 mL glass vial, sealed with a crimp cap (Agilent), and run using HS-GC/ECD. The samples were stabilized at 60 °C for 30 min in HS. Conditions were based on the report by Prabhu.^4^ After the stabilization, the aldehyde measurements were performed by GC/ECD using an HP-5 capillary column, 50 m × 0.32 mm × 1.05 μm (J&W Scientific, Folsom, CA). Helium (5.6) was the carrier gas at a flow rate of 1.0 mL/min, and for ECD nitrogen makeup, gas was applied at a flow rate of 30 mL/min. For calibration, a stock solution containing formaldehyde, acetaldehyde, and furfural (Sigma-Aldrich) in water was prepared separately for each compound in a concentration of 1000 mg/L. Calibration solutions were prepared in water ranging from 5–40 μg/L, 1.0–40 μg/L, and 0.5–25 μg/L for formaldehyde, acetaldehyde, and furfural, respectively. For each sample batch new calibration curves were made.

#### 2.3.2 GC/MSD

The acetaldehyde and furfural content were determined from FPBO water extract by using an Agilent 7890A gas chromatography instrument combined with an Agilent 5977B mass selective detector (GC/MSD). The split injection in a ratio of 1:10 was used. Compounds were released from the injector and kept at 250 °C for separation applying a J&W HP-INNOWax high polarity fused silica capillary column (length: 60 m, inner diameter: 0.25 mm, and film thickness: 0.25 μm) by using a carrier gas (helium 5.6) flow of 1.2 mL/min. The oven temperature program was as follows: initial temperature of 60 °C was held for 1 min, then the column was heated to 230 °C at 3 °C/min, and kept at this temperature for 30 min. The transfer line between GC/MS was kept at 280 °C. The compound detection with mass scan range of *m*/*z* between 27 and 300 (EI 70 eV) was used. The temperatures of the ionization source and quadrupole were 230 and 150 °C, respectively. For the analysis, 1 g of well homogenized FPBO was weighed and extracted in an ultrasonic bath with 20 mL of water. After extraction, the sample was centrifuged and 9 mL of water extract was mixed with 1 mL of internal standard (1-butanol, 1 g/L). Before GC/MSD analysis the sample was filtrated using a PTFE membrane filter (VWR) with a pore size of 0.45 μm to remove solid material. For the quantification, calibration solutions of acetaldehyde and furfural in the range of 20–420 mg/L and 10–200 mg/L were prepared, respectively. Integration of the peak areas for quantification was performed using target ions *m*/*z* 29, 43, 44, and 42 for acetaldehyde and target ions *m*/*z* 96, 95, 67, and 39 for furfural, respectively. Hence, the compounds can be selectively separated from other compounds that are eluted at the same retention time region.

#### 2.3.3 HPLC

For the HPLC analysis of the aldehydes (formaldehyde and acetaldehyde) and furfural, water extractions were performed in triplicate. The samples were taken from the freezer and allowed to come to room temperature for 3 h. The containers were then vigorously shaken for 10 min to ensure completely homogeneous samples. For the extraction, 1 g of FPBO was mixed with 40 g of water in a centrifuge tube and left for 24 h at room temperature. After 24 h, the water mixtures were centrifuged for 3 h at 4500 rpm to obtain clear water layers. Formaldehyde and acetaldehyde were analyzed based on the EPA Method 8315A. Accurately, 0.5 g of the water extract was taken and added in a 250 mL Florence flask. Thereafter, 100 mL of water, 4 mL of citrate buffer (citric acid and sodium citrate tribasic dihydrate from Sigma-Aldrich 99%), and 6 mL of DNPH reagent (99% 2,4-dinitrophenylhydrazine from Sigma-Aldrich and HPLC grade acetonitrile from Boom BV) was added, and the mixture was kept in an orbital shaker (VWR) at 40 °C for 1 h. Agitation was set to a gentle swirl. Immediately after 1 h, 10 mL of saturated NaCl (Merck 98%) was added. The DNPH derivatives were concentrated by use of an SPE setup with C18 cartridges (Thermo Scientific Hypersep C18 2000 mg). The derivatives were flushed off the SPE cartridges with 10 mL of acetonitrile and collected in test tubes. The acetonitrile weight was recorded. The DNPH derivatives were analyzed using a Hewlett-Packard 1100 series HPLC with a DAD detector set at 360 nm. Exactly 5 μL of sample was injected on a 250 × 4.6 mm 5 μm Agilent ZORBAX Eclipse XDB-C18 column at 30 °C. A gradient of acetonitrile/water was used as eluent starting with 65:35 for 15 min, 100:0 at 30 min, and 65:35 at 45 min and held for 15 min with a flow of 1 mL/min. The HPLC system was calibrated using a commercial standard of DNPH carbonyl derivatives (AccuStandard) diluted to 5 standards in a range of 10 to 50 mg/kg (concentration represented as nonderivatized carbonyl).

Furfural was analyzed by HPLC using the NREL/TP-510-42623 method. The water extracts were directly used for analysis. An Agilent 1200 series HPLC with VWD detector at 210 nm was used. A total of 5 μL of extract was injected on to a 300 × 7.8 mm 9 μm Bio-Rad Aminex HPX-87H column set at 60 °C. H_2_SO_4_ (5 mM) in water was used as eluent, and the flow rate was set to 0.55 mL/min. The furfural (Sigma-Aldrich 99%) response was calibrated using 6 freshly prepared standards in a range of 25 to 250 mg/L.

#### 2.3.4 UV/Vis Spectrometry

The formaldehyde content was determined by UV/Vis spectrometry at 412 nm after formation of the complex diacetyldihydrolutidine. For the analysis, 0.16 g of FPBO was extracted with 50 mL of water in a 100 mL volumetric flask by gently swirling for 5 min before and after the ultrasonic bath treatment for 5 min at ambient temperature. After extraction, the volumetric flask was filled up to mark with water and filtered using a polyethersulfon syringe filter (0.45 μm Fisherbrand). For the analysis, 0.5 mL of the filtered extract was pipetted into a 50 mL volumetric flask and mixed with 5.0 mL of reagent solution of acetylacetone and water. The acetyl acetone reagent solution was prepared by mixing 15 g of anhydrous ammonium acetate (Sigma-Aldrich), 0.3 mL of glacial acetic acid (Sigma-Aldrich), and 0.2 mL of acetylacetone (Sigma-Aldrich) reagent with water in a 100 mL flask. The sample reference solution was prepared by pipetting 0.5 mL of the filtered sample extract and a 5.0 mL of reagent solution without acetyl acetone and water in a 100 mL volumetric flask. The reagent solution without acetyl acetone was prepared by mixing 15 g of anhydrous ammonium acetate (Sigma-Aldrich) and 0.3 mL of glacial acetic acid (Sigma-Aldrich) reagent with water in a 50 mL volumetric flask. For the calibration, formaldehyde standards ranging from 0 to 0.370 mg/L were prepared by pipetting 0, 0.4, 1.0, and 2.0 mL of formaldehyde stock solution of 9.25 mg/L and 5 mL of acetylacetone reagent solution, same as used for the samples, into a volumetric flask of 50 mL and filled with water to the mark. Prior to analysis, the samples and standards were shaken for at least 15 s and immersed (whole flask with cap) in a thermostatic water bath set at 60 °C for 10 min, followed by cooling for 2 min in a cooling bath (0 °C). After cooling, the flasks were shaken again for at least 15 s. Absorbance measurements at 412 nm (λ of max. absorbance) were performed between 35 and 60 min from the time when the flasks were placed in the heated water bath. Absorbance measurements of the standard solutions were performed against water, and the sample solutions were measured against their sample reference solution. The solutions were measured by applying a double-beam PerkinElmer, Lambda 25, UV/Vis spectrophotometer and applying the UV WinLab V6.0 software package.

### 2.4 Statistical Comparison of Methods

For each analyte, three analysis methods were used to determine the concentration. A two-way Analysis of Variance (two-way ANOVA) was conducted to analyze the effect of the three analytical methods and the five types of samples on the concentration of the analyte. IBM SPSS statistics version 25 was used to conduct the two-way ANOVA with a significance level of 0.05.

## 3 Results and Discussion

### 3.1 Comparison of Methods (HS-GC/ECD, HPLC, UV/Vis, GC/MSD)

The formaldehyde analysis was performed using three different methods at three different laboratories (VTT, RUG, and BTG). The methods used applied derivatization with PFBHA, DNPH, or acetylacetone/ammonium acetate reagents to form a formaldehyde complexes, which were analyzed by HS-GC/ECD, HPLC, and UV/Vis-spectroscopy, respectively. Acetaldehyde was the second aldehyde, whose content was determined from the FPBOs using three different methods. In this case, acetaldehyde was measured by HS-GC/ECD and HPLC, applying PFBHA and DNPH derivatization as well direct analysis by GC/MSD of the water extract. For the furfural analysis the same methods were used as for the acetaldehyde analysis, except HPLC analysis was performed directly from water extract.

Only with the HS-GC/ECD method could all studied compounds be analyzed simultaneously, whereas UV/Vis was suitable for formaldehyde and GC/MSD for acetaldehyde and furfural determination. Different HPLC methods were used to determine aldehydes (formaldehyde and acetaldehyde) and furfural, in which the former was determined as a DNPH derivative and the latter analyzed directly from a water extract. It has been reported that DNPH derivatization followed by HPLC-UV analysis is not suitable for the quantitative analysis of aldehydes from FPBOs, due to the side reactions of reagents and FPBO components.^5^ In this study disturbances of the matrix were eliminated by first extracting the FPBO with water and using the extract for derivatization. The weakness of the UV/Vis method is that it is only suitable for the formaldehyde analysis; however, it is rather easy to perform in comparison to the other derivatization methods. Oppositely, direct GC/MSD analysis of water extract of FPBO cannot be used to determine formaldehyde, because in water it is mainly present as methylene glycol.^13^ Direct GC/MSD analysis of water extracts enables the determination of various low molecular weight water-soluble FPBO compounds simultaneously, also including compounds other than aldehydes.^17^ Additional advantages of the HPLC method is that simultaneously with aldehyde formaldehyde and acetaldehyde analysis the total content of carbonyls can be determined. For the method comparison the detector linearity was evaluated by determining the correlation coefficient (R2) of linear regression analysis of the calibration curve constructed between the peak area and analyte concentration for each compound in different methods (Table 3).

**Table 3:** Equation Chart, R2, Limit of Detection (LOD), Limit of Quantification (LOQ), and Residual Standard Deviation (RSD)% for Formaldehyde, Acetaldehyde, and Furfural Analysis Methods

method	calibration curve	R2	linear range	LOD (S/N 3:1)^a^^,^^b^	LOQ S/N (10:1)^a^^,^^c^	RSD% (*n* = 15)
Formaldehyde						
HS-GC/ECD	*y* = 18136900*x* + 3818	0.9989	5–40 μg/L	n.d.	n.d.	4.0
HPLC	*y* = 690.6*x* + 145.8	0.9997	10–30 μg/L	0.0049 μg/L	0.016 μg/L	5.0
UV/Vis	*y* = 0.2804*x* + 0.0013	0.9999	0–0.37 mg/L	n.d.	n.d.	1.4
Acetaldehyde						
HS-GC/ECD	*y* = 4367*x* + 4349	0.9991	1.0–40 μg/L	n.d.	n.d.	6.1
GC/MSD	*y* = 28235*x* + 35471	0.9998	20–420 mg/L	1.5 mg/L	5.0 mg/L	7.3
HPLC	*y* = 555.9*x* + 78.8	0.9997	5–20 μg/L	0.003 μg/L	0.01 μg/L	14
Furfural						
HS-GC/ECD	*y* = 12163*x* – 2960	0.9998	0.5–25 μg/L	n.d.	n.d.	7.2
GC/MSD	*y* = 217852*x* + 957028	0.9998	10–200 mg/L	2.2 mg/L	7.1 mg/L	2.2
HPLC	*y* = 246.7*x* + 171.6	1.0000	25–200 mg/L	0.011 mg/L	0.036 mg/L	5.5

a n.d. = not determined.

b LOD = limit of detection; S/N signal noise ratio of 3:1.

c LOQ = limit of quantification; S/N signal noise ratio of 10:1.

The linear range and limit of quantitation (LOQ) varied among the HPLC and GC/MSD methods. For the HS-GC/ECD and UV/Vis methods the LOQ values were not determined. The ECD is a halogen selective detector having a much higher sensitivity than a FID or MSD. Combination of aldehyde selective halogenated derivatization reagent, and the halogen selective detector enhances sensitivity of the method significantly. For the analysis of oxime derivatives in the linear range of the detector (ECD) about 5000 to 10 000 times dilution was needed for the samples. A similar dilution was performed for the sample prior analysis of DNPH derivatives and diacetyldihydrolutidine formaldehyde complexes by HPLC and UV/Vis, respectively. Thus, much lower concentration can be measured by the derivatization methods than that was present in the studied samples. In addition, limit values set for REACH (Table 1) can be well achieved. The lowest sensitivity was obtained with direct GC/MSD analysis after water extraction of FPBOs. Integration of acetaldehyde and furfural peak areas was done using target ions that improve sensitivity, and interference of compounds eluted in similar regions was minimized. The R2 was good for all compounds and the methods studied. Residual standard deviation for three parallel analyses for each sample remained below 10%, exception acetaldehyde was determined by HPLC after DNPH derivatization.

The results of formaldehyde, acetaldehyde, and furfural analysis with the methods studied from different FPBOs and average concentrations determined in different methods from the same sample are presented in the Table 4. Concentrations of formaldehyde, acetaldehyde, and furfural measured with different methods followed the same order among different samples, except formaldehyde in pine after vacuum evaporation and furfural in bark and miscanthus. The individual values of the parallel measurements of each sample were compared with a two-way ANOVA tests (Section 3.2).

**Table 4:** Concentrations (wt % on a Wet Basis) of Formaldehyde, Acetaldehyde, and Furfural Determined by Different Methods and the Average of the Three Methods

wt % ± sd *n* = 3^a^	pine, aged	pine, filtrated	pine, vacuum evaporated	miscanthus	bark
Formaldehyde^b^					
HS-GC/ECD	1.10 ± 0.01	1.76 ± 0.12	1.52 ± 0.02	0.83 ± 0.06	1.44 ± 0.05
HPLC	1.27 ± 0.04	1.85 ± 0.30	1.90 ± 0.25	1.05 ± 0.03	1.49 ± 0.06
UV/Vis	1.25 ± 0.00	1.84 ± 0.01	1.84 ± 0.02	0.84 ± 0.02	1.35 ± 0.04
average	1.21 ± 0.09	1.82 ± 0.05	1.75 ± 0.20	0.91 ± 0.12	1.43 ± 0.07
Acetaldehyde					
HS-GC/ECD	0.08 ± 0.00	0.30 ± 0.01	0.02 ± 0.00	0.44 ± 0.01	0.60 ± 0.01
GC/MSD	0.08 ± 0.00	0.32 ± 0.01	0.02 ± 0.00	0.45 ± 0.02	0.59 ± 0.09
HPLC	0.15 ± 0.04	0.24 ± 0.04	0.06 ± 0.01	0.38 ± 0.01	0.53 ± 0.04
average	0.10 ± 0.04	0.29 ± 0.04	0.03 ± 0.02	0.42 ± 0.04	0.57 ± 0.04
Furfural					
HS-GC/ECD	0.18 ± 0.01	0.21 ± 0.03	0.11 ± 0.01	0.30 ± 0.03	0.23 ± 0.02
GC/MSD	0.20 ± 0.00	0.23 ± 0.01	0.15 ± 0.01	0.34 ± 0.00	0.28 ± 0.00
HPLC	0.24 ± 0.01	0.32 ± 0.02	0.21 ± 0.03	0.36 ± 0.01	0.36 ± 0.00
average	0.21 ± 0.03	0.25 ± 0.06	0.16 ± 0.05	0.33 ± 0.03	0.29 ± 0.07

a sd = standard deviation. *n* = number of parallel measurements.

b Measured concentration is the sum of formaldehyde and methylene glycol; see Section 3.3.

### 3.2 Comparison of Results (Formaldehyde, Acetaldehyde, and Furfural)

At first glance the results from the three different methods per analyte appear to be similar. A two-way Analysis of Variance with a significance level of 0.05 is used per analyte to determine if the analytical methods used are statistically different. The null hypothesis is that there is no significant effect in the determination of concentration across the three methods (H_0_:μ_method1_ = μ_method2_ = μ_method3_). The triplicate data of all five samples were used in performing the two-way ANOVA. The effect of FPBO sample types were also determined but were not further explained because of the obvious reason that sample types will result in different concentrations. The output of the two-way ANOVA results is shown in Table 5 for formaldehyde, 7 for acetaldehyde, and 8 for furfural.

**Table 5:** Two-Way ANOVA Results for the Analysis of Formaldehyde

source of variation	SS	df	MS	*F*	*P*-value
FPBO samples	5.180	4	1.295	107.368	0.000
analytical method	0.253	2	0.126	10.469	0.000
interaction	0.182	8	0.023	1.882	0.100
error	0.362	30	0.012		
total	97.029	45			

#### 3.2.1 Formaldehyde

A two-way ANOVA (Table 5) revealed that there was not a statistically significant interaction between the effects of FPBO types and analysis methods (*p* = 0.100). The main effect for FPBO sample types indicates a statistically significant difference on the concentration of formaldehyde (*p* = 0.000). The main effect for the analytical method also indicates a statistically significant difference on the concentration of formaldehyde (*p* = 0.000). The null hypothesis is rejected.

A Tukey’s HSD Test for the main effect of the analytical methods was performed, and the results are given in Table 6. Multiple comparisons found that the mean concentrations were significantly different between the HPLC and HS-GC/ECD method (*p* = 0.000). There was no statistically significant difference in mean concentration between the UV/vis and HPLC (*p* = 0.096) or between UV/vis and HS-GC/ECD (*p* = 0.055).

**Table 6:** Multiple Comparisons of Methods for the Analysis of Formaldehyde Using the Tukey HSD^a^

					95% confidence interval	
(I) method	(J) method	mean difference (I – J)	std. error	sig.	lower bound	upper bound
HPLC	HS-GC/ECD	0.183^b^	0.04	0.000	0.085	0.282
	UV	0.086	0.04	0.096	–0.013	0.185
HS-GC/ECD	HPLC	–0.183^b^	0.04	0.000	–0.282	–0.085
	UV/Vis	–0.097	0.04	0.055	–0.196	0.002
UV/Vis	HPLC	–0.086	0.04	0.096	–0.185	0.013
	HS-GC/ECD	0.097	0.04	0.055	–0.002	0.196

a Based on observed means. The error term is mean square (error) = 0.012.

b The mean difference is significant at the 0.05 level.

#### 3.2.2 Acetaldehyde

A two-way ANOVA (Table 7) revealed that there is a statistically significant interaction between the effects of FPBO types and analysis methods (*p* = 0.001). The main effect for FPBO sample types indicates a statistically significant difference on the concentration of acetaldehyde (*p* = 0.000). The main effect for the analytical method indicates no statistically significant difference on the concentration of acetaldehyde (*p* = 0.232). The null hypothesis is accepted. No Tukey’s HSD Test was performed for the analysis of acetaldehyde.

**Table 7:** Two-Way ANOVA Results for the Analysis of Acetaldehyde

source of variation	SS	df	MS	*F*	*P*-value
FPBO samples	1.789	4	0.447	445.482	0.000
analytical method	0.003	2	0.002	1.532	0.232
interaction	0.039	8	0.005	4.856	0.001
error	0.030	30	0.001		
total	5.483	45			

#### 3.2.3 Furfural

A two-way ANOVA (Table 8) revealed that there is a statistically significant interaction between the effects of FPBO types and analysis methods (*p* = 0.001). The main effect for FPBO sample types indicates a statistically significant difference on the concentration of furfural (*p* = 0.000). The main effect for the analytical method also indicates a statistically significant difference on the concentration of furfural (*p* = 0.000). The null hypothesis is rejected.

**Table 8:** Two-Way ANOVA Results for the Analysis of Furfural

source of variation	SS	df	MS	*F*	*P*-value
FPBO samples	0.175	4	0.044	194.186	0.000
analytical method	0.071	2	0.036	158.124	0.000
interaction	0.008	8	0.001	4.681	0.001
error	0.007	30	0.000		
total	3.034	45			

A Tukey’s HSD Test for the main effect of the analytical methods was performed. Multiple comparisons found that the mean concentration was significantly different between all three methods with identical *p*-values (*p* = 0.000) (Table 9).

**Table 9:** Multiple Comparisons of Methods for the Analysis of Furfural Using the Tukey HSD^a^

					95% confidence interval	
(I) method	(J) method	mean difference (I – J)	std. error	sig.	lower bound	upper bound
GC/MSD	HPLC	–0.058^b^	0.01	0.000	–0.072	–0.045
	HS-GC/ECD	0.380^b^	0.01	0.000	0.025	0.052
HPLC	GC/MSD	0.058^b^	0.01	0.000	0.045	0.072
	HS-GC/ECD	0.097^b^	0.01	0.000	0.083	0.110
HS-GC/ECD	GC/MSD	–0.038^b^	0.01	0.000	–0.052	–0.025
	HPLC	–0.097^b^	0.01	0.000	–0.110	–0.083

a Based on observed means. The error term is mean square (error) = 0.000.

b The mean difference is significant at the 0.05 level.

### 3.3 Effect of Feedstocks, Storage, and After Treatments on Aldehydes and Furfural Concentration

The effect of the vacuum evaporation and room temperature storage for one year on aldehydes and furfural concentration in FPBO from pine was evaluated based on the results obtained by different methods. In addition, comparison of different feedstocks (pine, bark, and miscanthus) was made. All results are presented in Table 4.

In aqueous solutions, formaldehyde is mainly present as methylene glycol^13^ and might react further to form dimers.^15^ The FPBOs are acidic liquids (∼pH 3) containing about 15–25 wt % of water^2^ but also a small quantity of alcohols such as methanol.^8^ Alcohols like methanol are used to prevent further polymerization reactions of formaldehyde in aqueous solutions.^18^ The same phenomenon might take place in FPBOs. Based on these facts, it is expected that the major part of the formaldehyde in the FPBO is present as methylene glycol and derivatives thereof. It has been shown that dehydration of methylene glycol will occur when formaldehyde is continuously removed from the aqueous solution.^14^ In analysis it is expected that both components (formaldehyde and methylene glycol) are converted to complexes by using the derivatization methods. Therefore, the formaldehyde concentration measured in the FPBO is actually the sum of formaldehyde and methylene glycol. Thus, from this point on formaldehyde and methylene glycol (and oligomers) will be referred to as FAMG.

Vacuum evaporation followed by filtration did not affect the formaldehyde concentration in FPBO from pine when compared to filtration only. This result clearly supports the theory that in the FPBO formaldehyde is in the form of methylene glycol and/or oligomers. The boiling point of methylene glycol (194 °C at 101 kPa) is much higher than that of formaldehyde (−19 °C). The room temperature storage of FPBO reduced the formaldehyde concentration, which strongly indicates the occurrence of irreversible reactions of formaldehyde or its hydrated form with other in FPBO present components (such as phenolics). The feedstocks processed in pyrolysis have different chemical compositions^19,20^ that also affect the composition of volatiles formed in fast pyrolysis. The decreasing order of formaldehyde in FPBOs from different feedstock was pine > bark > miscanthus.

Vacuum evaporation and long-term room temperature storage in a closed container decreased the acetaldehyde concentration in pine derived FPBO when compared to the filtration only. Obviously, acetaldehyde is released during the vacuum evaporation due to its volatility and because in the aqueous solution it is mainly present in the nonhydrated form,^21^ whereas in the room temperature storage acetaldehyde may react with other FPBO components. Similar behavior of acetaldehyde and formaldehyde at long-term room temperature storage was observed. In the vacuum evaporation acetaldehyde was almost completely removed in contrast to formaldehyde. These results also support that a major part of formaldehyde in FPBOs is present as methylene glycol and cannot be removed readily by evaporation, whereas acetaldehyde does not convert in water to diols so easily. Because of the low boiling point, it can be easily evaporated.

Based on the results, the feedstock type only has a small influence on the acetaldehyde concentration in FPBOs. The highest concentration was detected in the FPBO produced from bark.

Compared to the aldehydes studied, furfural is only partly soluble in water. Nevertheless, furfural concentrations were determined using the same methods as applied for acetaldehyde. After storage only a minor decrease in furfural concentration was observed. The highest difference in furfural concentration before and after storage was observed with the HPLC method. Based on the HS-GC/ECD, one year of storage did not affect the furfural concentration, whereas GC/MSD showed a minor decrease. Further studies are needed to confirm the effect of storage on the furfural concentration. However, the overall change in the furfural concentration in long-term storage was smaller in comparison to formaldehyde and acetaldehyde. Furfural has been found to polymerize and convert to smaller molecules in an acidic environment.^22−24^ However, the reaction is much slower compared to the aliphatic aldehydes. About half of the furfural was removed by vacuum evaporation. This was less when compared to acetaldehyde but much more compared to the formaldehyde, which was obviously not reduced by the vacuum evaporation.

The furfural concentration in FBPOs made from different feedstocks varied slightly. Furfural is produced by the degradation of C-5 sugars in polysaccharides, and thus the fraction of polysaccharides in the biomass affects the amount of furfural formed.^25^

## 4 Conclusions

Different analysis methods to determine the concentration of acetaldehyde, formaldehyde, and furfural in FPBO were evaluated. The three methods for the determination of formaldehyde and furfural differ significantly (*p* = 0.000), whereas the three methods for the determination of acetaldehyde have no significant difference (*p* = 0.232). The accuracy of the methods could not be determined due to the lack of a reference sample, and therefore no statement can be made about the trueness of the methods. The precisions of all methods were good with RSDs below 10%, with the exception of the HPLC analysis of acetaldehyde with an RSD of 14%. As a future action, the accuracies of different methods need to be studied.

The feedstock type, aging at room temperature, and pretreatment affected the aldehydes and furfural concentration. The highest concentrations of formaldehyde and acetaldehyde were determined from pine and bark, respectively. A similar concentration of furfural was in all the FPBOs from pine, miscanthus, and bark. The concentration of formaldehyde was the highest of all compounds in all studied samples followed by acetaldehyde and furfural, respectively.

Aging altered the concentration of all the compounds. The most significant decreases were observed for the acetaldehyde and formaldehyde concentrations, and the decrease was only minor for furfural. The decrease was proposed to be the result of chemical reactions of acetaldehyde and furfural with other compounds present in the FPBO and/or as result of the self-condensation reactions. Vacuum evaporation as a pretreatment method released acetaldehyde almost completely from the FPBO and about half of the furfural content. However, the formaldehyde concentration before and after vacuum evaporation was the same. The results indicated that, in the FPBO, chemical equilibrium between formaldehyde and methylene glycol exists similar to in the aqueous phase. In the analysis, both components (formaldehyde and methylene glycol) are converted to complexes by using the derivatization methods. Therefore, the formaldehyde concentration measured in the FPBO is actually the sum of formaldehyde and methylene glycol. It is suggested that formaldehyde and methylene glycol will be in the future referred to as FAMG.

## Supporting Information Available

Examples of UV/Vis spectra, gas chromatogram from HS-GC/ECD, total ion chromatogram from GC/MSD, chromatograms from HPLC analysis for formaldehyde, acetaldehyde, and furfural analyses, three SPSS outputs from formaldehyde, and acetaldehyde output two-way ANOVA Tukey (PDF)
