# Supplementary material for: Evaluation of Analysis Methods for Formaldehyde, Acetaldehyde, and Furfural from Fast Pyrolysis Bio-oil
Source: Energy Fuels. 2021 Oct 29;35(22):18583–91. doi: 10.1021/acs.energyfuels.1c02208 (PMC8607318; doi:10.1021/acs.energyfuels.1c02208)
Supplement: Supplementary file 1 — ef1c02208_si_001.pdf [file ef1c02208_si_001.pdf]

Evaluation of analysis methods for formaldehyde, acetaldehyde, and furfural from fast pyrolysis bio-oil

Taina Ohra-aho[1], Léon Rohrbach[2], Jozef G.M. Winkelman[2], Hero J. Heeres[2], Atte Mikkelson[1], Anja Oasmaa[1], Bert van de Beld[3], Evert. J. Leijenhorst[3], Hans Heeres[3]*

[1]VTT Technical Research Centre of Finland Ltd, P.O. Box 1000, FI-02044 VTT, Finland

[2]Dept. of Chemical Engineering (ENTEG), University of Groningen, Nijenborgh 4, 9747 AG Groningen, The Netherlands

[3]BTG Biomass Technology Group BV, P.O. Box 835, 7500 AV Enschede, The Netherlands

***Corresponding Author:** Taina Ohra-aho, taina.ohra-aho@vtt.fi

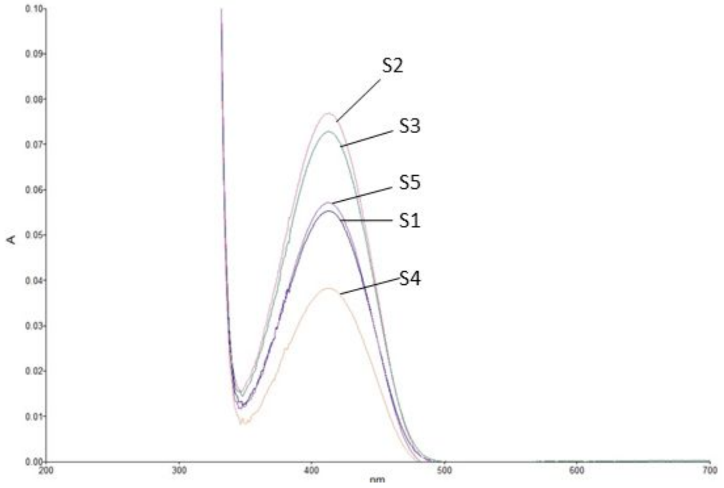

Figure S1. Spectra of pine, aged (S1); pine, filtrated (S2); pine, vacuum-evaporated (S3); miscanthus (S4); bark (S5) samples measured at 412 nm for formaldehyde analysis as diacetyldihydrolutidine complex.

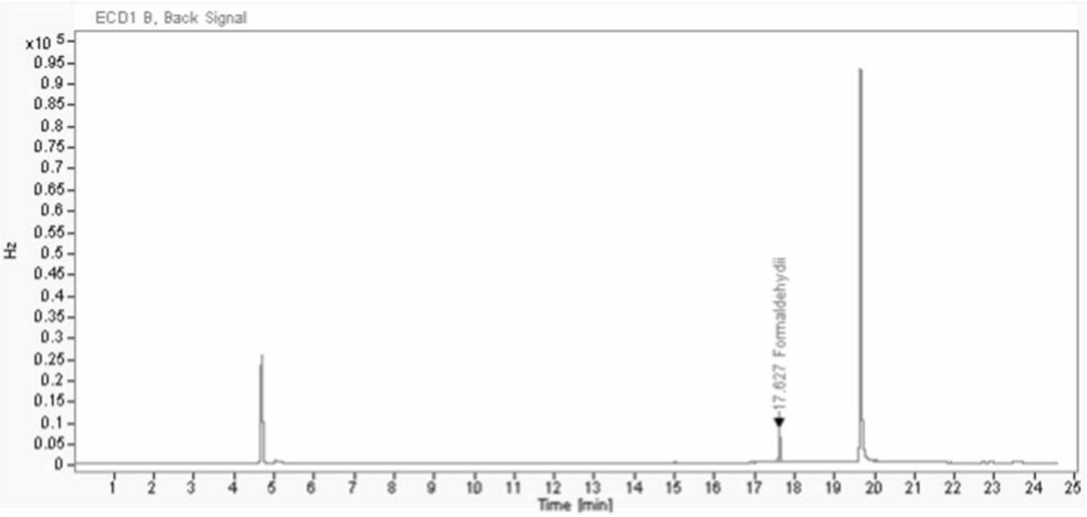

Figure S2. Gas chromatogram obtained after StHS-GC/ECD analysis of formaldehyde-oxime from FPBO miscanthus.

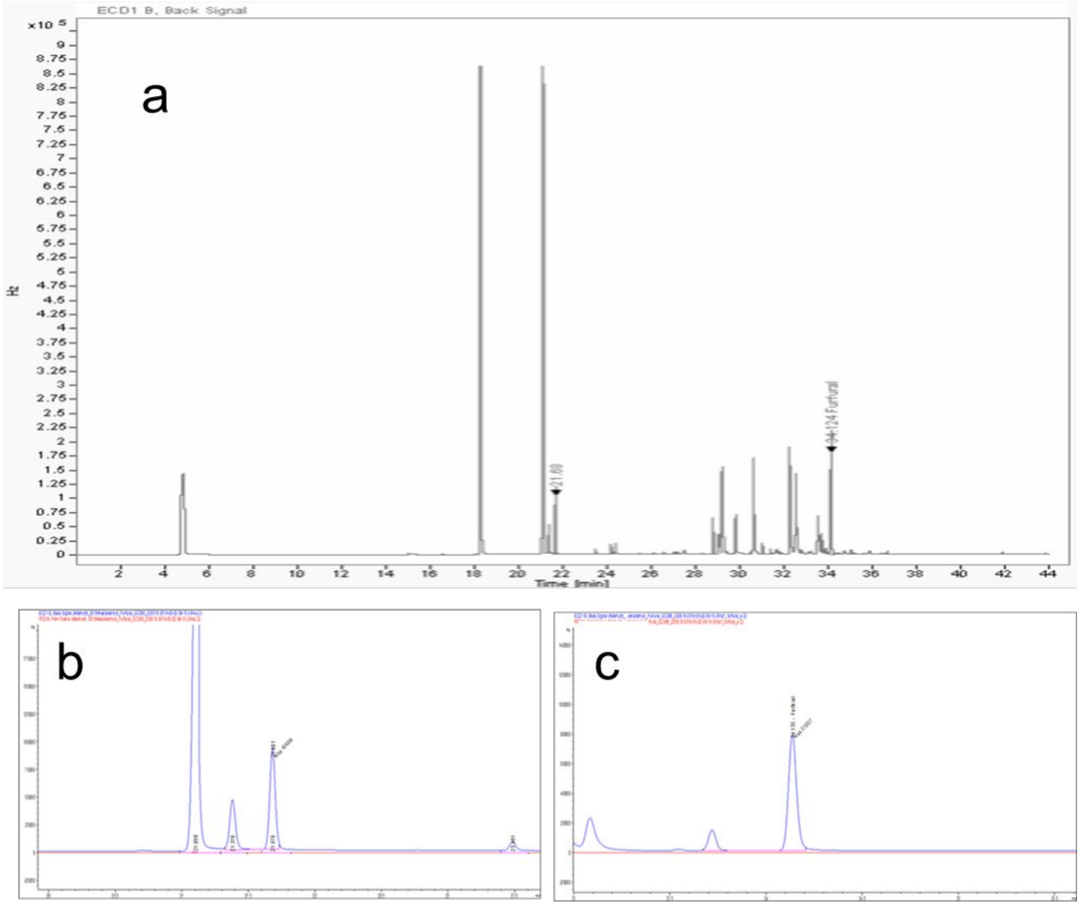

Figure S3. Gas chromatogram obtained after StHS-GC/ECD analysis of acetaldehyde (rt 21.68) and furfural (rt 34.124) as oximes from FPBO from miscanthus. Whole gas chromatogram of sample (a); two isomers (peaks) of aldehyde –oxime (b); two isomers (peaks) of furfural-oxime (c)

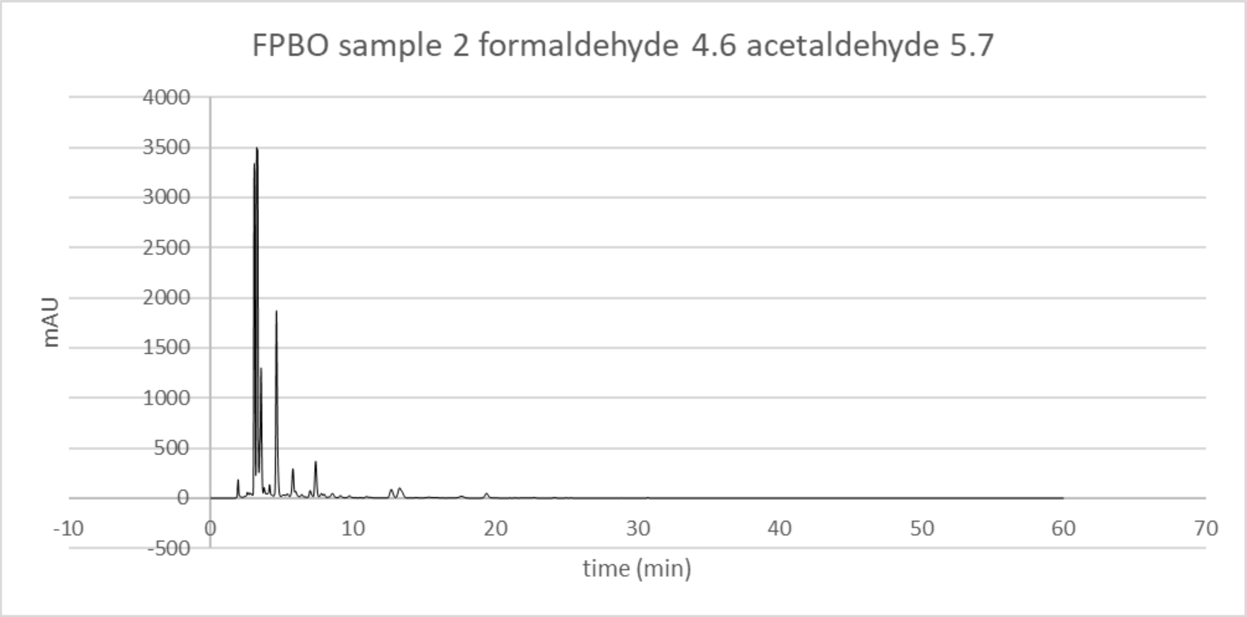

Figure S4. HPLC chromatogram of 2,4-dinitrophenylhydrazones of formaldehyde (rt 4.6) and acetaldehyde (rt 5.7) in FPBO from pine, filtrated

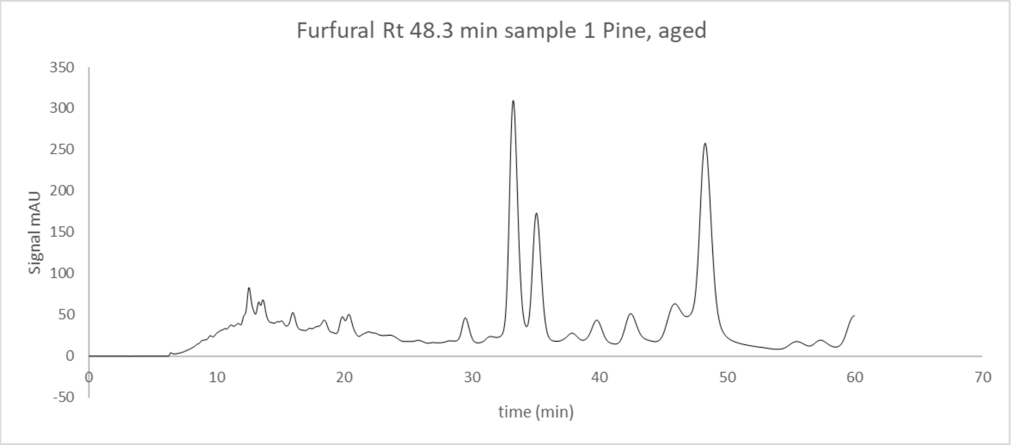

Figure S5. HPLC chromatogram of furfural obtained from water extract of FPBO from pine, aged.

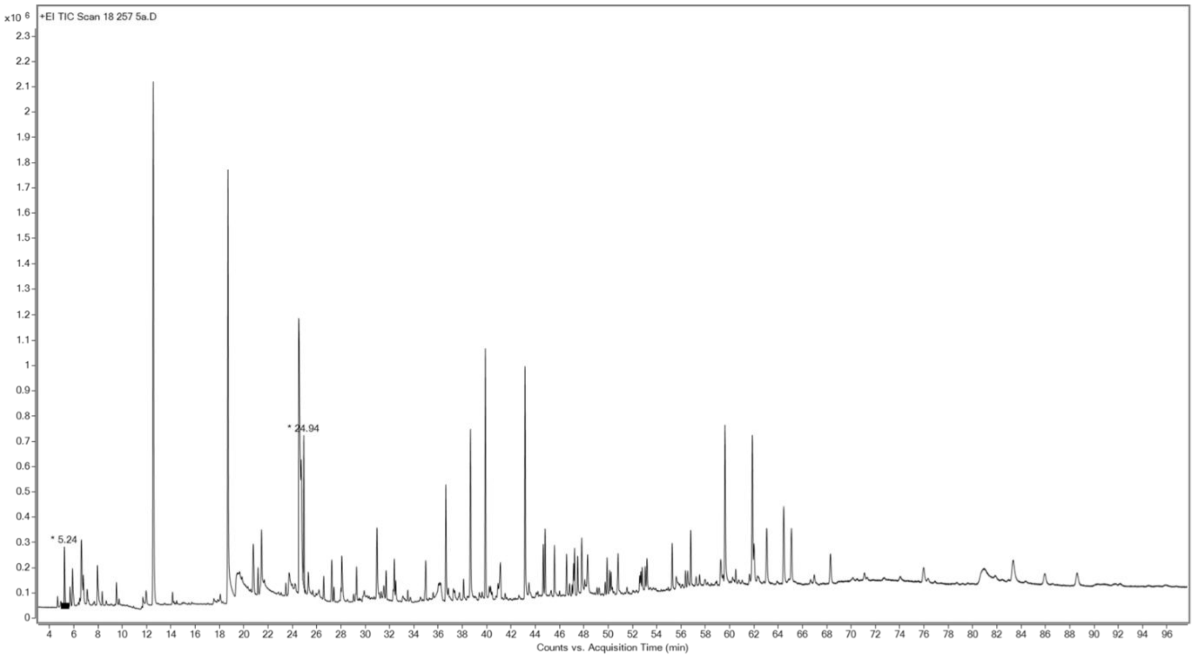

Figure S6. Total ion chromatogram of FPBO from bark after water extraction: rt 5.24 acetaldehyde; rt 24.94 furfural.

Univariate Analysis of Variance Formaldehyde

Between-Subjects Factors

		N
sample	1	9
	2	9
	3	9
	4	9
	5	9
method	HPLC	15
	HS-GC-ECD	15
	UV	15

Descriptive Statistics

Dependent Variable: concentration

sample	method	Mean	Std. Deviation	N
1	HPLC	1.271	0.040	3
	HS-GC-ECD	1.103	0.013	3
	UV	1.253	0.006	3
	Total	1.209	0.083	9
2	HPLC	1.851	0.298	3
	HS-GC-ECD	1.757	0.124	3
	UV	1.840	0.010	3
	Total	1.816	0.167	9
3	HPLC	1.898	0.250	3
	HS-GC-ECD	1.523	0.021	3
	UV	1.840	0.030	3
	Total	1.754	0.216	9
4	HPLC	1.052	0.033	3
	HS-GC-ECD	0.827	0.055	3
	UV	0.843	0.015	3
	Total	0.907	0.113	9
5	HPLC	1.490	0.059	3
	HS-GC-ECD	1.435	0.051	3
	UV	1.353	0.029	3
	Total	1.426	0.073	9
Total	HPLC	1.512	0.370	15
	HS-GC-ECD	1.329	0.343	15
	UV	1.426	0.392	15
	Total	1.422	0.369	45

Levene's Test of Equality of Error Variances[a,b]

		Levene Statistic	df1	df2	Sig.
conc	Based on Mean	6.859	14	30	.000
	Based on Median	1.281	14	30	.275
	Based on Median and with adjusted df	1.281	14	5.070	.419
	Based on trimmed mean	6.153	14	30	.000

Tests the null hypothesis that the error variance of the dependent variable is equal across groups.

a. Dependent variable: conc

b. Design: Intercept + sample + method + sample * method

Tests of Between-Subjects Effects

Dependent Variable: conc

Source	Type III Sum of Squares	df	Mean Square	F	Sig.	Partial Eta Squared
Corrected Model	5.614[a]	14	.401	33.245	.000	.939
Intercept	91.053	1	91.053	7548.225	.000	.996
sample	5.180	4	1.295	107.358	.000	.935
method	.253	2	.126	10.469	.000	.411
sample * method	.182	8	.023	1.882	.100	.334
Error	.362	30	.012			
Total	97.029	45				
Corrected Total	5.976	44				

a. R Squared = .939 (Adjusted R Squared = .911)

Estimated Marginal Means

1. Grand Mean

Dependent Variable: conc

Mean	Std. Error	95% Confidence Interval	
		Lower Bound	Upper Bound
1.422	.016	1.389	1.456

2. sample

Estimates

Dependent Variable: conc

sample	Mean	Std. Error	95% Confidence Interval	
			Lower Bound	Upper Bound
1	1.209	.037	1.134	1.284
2	1.816	.037	1.741	1.891
3	1.754	.037	1.679	1.828
4	.907	.037	.832	.982
5	1.426	.037	1.352	1.501

Pairwise Comparisons

Dependent Variable: conc

(I) sample	(J) sample	Mean Difference (I-J)	Std. Error	Sig.[b]	95% Confidence Interval for Difference[b]	
					Lower Bound	Upper Bound
1	2	-.607*	.052	.000	-.764	-.450
	3	-.544*	.052	.000	-.701	-.388
	4	.302*	.052	.000	.145	.459
	5	-.217*	.052	.002	-.374	-.060
2	1	.607*	.052	.000	.450	.764
	3	.062	.052	1.000	-.094	.219
	4	.909*	.052	.000	.752	1.066
	5	.390*	.052	.000	.233	.547
3	1	.544*	.052	.000	.388	.701
	2	-.062	.052	1.000	-.219	.094
	4	.846*	.052	.000	.690	1.003
	5	.327*	.052	.000	.170	.484
4	1	-.302*	.052	.000	-.459	-.145
	2	-.909*	.052	.000	-1.066	-.752
	3	-.846*	.052	.000	-1.003	-.690
	5	-.519*	.052	.000	-.676	-.362
5	1	.217*	.052	.002	.060	.374
	2	-.390*	.052	.000	-.547	-.233
	3	-.327*	.052	.000	-.484	-.170
	4	.519*	.052	.000	.362	.676

Based on estimated marginal means

*. The mean difference is significant at the .05 level.

b. Adjustment for multiple comparisons: Bonferroni.

Univariate Tests

Dependent Variable: conc

	Sum of Squares	df	Mean Square	F	Sig.	Partial Eta Squared
Contrast	5.180	4	1.295	107.358	.000	.935
Error	.362	30	.012			

The F tests the effect of sample. This test is based on the linearly independent pairwise comparisons among the estimated marginal means.

3. method

Estimates

Dependent Variable: conc

method	Mean	Std. Error	95% Confidence Interval	
			Lower Bound	Upper Bound
HPLC	1.512	.028	1.454	1.570
HS-GC-ECD	1.329	.028	1.271	1.387
UV	1.426	.028	1.368	1.484

Pairwise Comparisons

Dependent Variable: conc

(I) method	(J) method	Mean Difference (I-J)	Std. Error	Sig.[b]	95% Confidence Interval for Difference[b]	
					Lower Bound	Upper Bound
HPLC	HS-GC-ECD	.183*	.040	.000	.082	.285
	UV	.086	.040	.118	-.015	.188
HS-GC-ECD	HPLC	-.183*	.040	.000	-.285	-.082
	UV	-.097	.040	.066	-.199	.005
UV	HPLC	-.086	.040	.118	-.188	.015
	HS-GC-ECD	.097	.040	.066	-.005	.199

Based on estimated marginal means

*. The mean difference is significant at the .05 level.

b. Adjustment for multiple comparisons: Bonferroni.

Univariate Tests

Dependent Variable: conc

	Sum of Squares	df	Mean Square	F	Sig.	Partial Eta Squared
Contrast	.253	2	.126	10.469	.000	.411
Error	.362	30	.012			

The F tests the effect of method. This test is based on the linearly independent pairwise comparisons among the estimated marginal means.

4. sample * method

Dependent Variable: conc

sample	method	Mean	Std. Error	95% Confidence Interval	
				Lower Bound	Upper Bound
1	HPLC	1.271	.063	1.142	1.401
	HS-GC-ECD	1.103	.063	.973	1.232
	UV	1.253	.063	1.124	1.383
2	HPLC	1.851	.063	1.721	1.980
	HS-GC-ECD	1.757	.063	1.628	1.887
	UV	1.840	.063	1.710	1.970
3	HPLC	1.898	.063	1.768	2.027
	HS-GC-ECD	1.523	.063	1.393	1.652
	UV	1.840	.063	1.710	1.970
4	HPLC	1.052	.063	.922	1.181
	HS-GC-ECD	.827	.063	.697	.956
	UV	.843	.063	.714	.973
5	HPLC	1.490	.063	1.361	1.620
	HS-GC-ECD	1.435	.063	1.306	1.565
	UV	1.353	.063	1.224	1.483

Post Hoc Tests method

Multiple Comparisons

Dependent Variable: conc

Tukey HSD

(I) method	(J) method	Mean Difference (I-J)	Std. Error	Sig.	95% Confidence Interval Lower Bound	Upper Bound
HPLC	HS-GC-ECD	.183404804303901*	.040104504052353	.000	.084536322669262	.282273285938539
	UV	.086391866274560	.040104504052353	.096	-.012476615360079	.185260347909198
HS-GC-ECD	HPLC	-.183404804303901*	.040104504052353	.000	-.282273285938539	-.084536322669262
	UV	-.097012938029341	.040104504052353	.055	-.195881419663980	.001855543605297
UV	HPLC	-.086391866274560	.040104504052353	.096	-.185260347909198	.012476615360079
	HS-GC-ECD	.097012938029341	.040104504052353	.055	-.001855543605297	.195881419663980

Based on observed means.

The error term is Mean Square(Error) = .012.

*. The mean difference is significant at the .05 level.

Homogeneous Subsets

conc

Tukey HSD[a,b]

method	N	Subset 1	Subset 2
HS-GC-ECD	15	1.328987061970659	
UV	15	1.426000000000000	1.426000000000000
HPLC	15		1.512391866274560
Sig.		.055	.096

Means for groups in homogeneous subsets are displayed.

Based on observed means.

The error term is Mean Square(Error) = .012.

a. Uses Harmonic Mean Sample Size = 15.000.

b. Alpha = .05.

Profile Plots

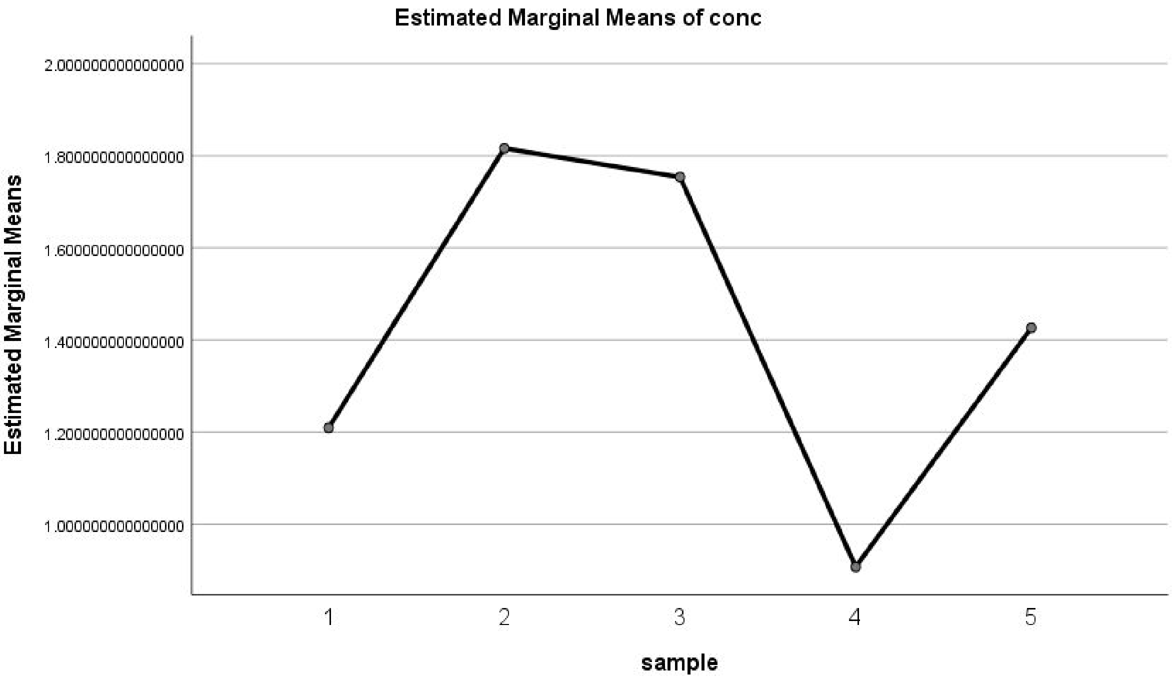

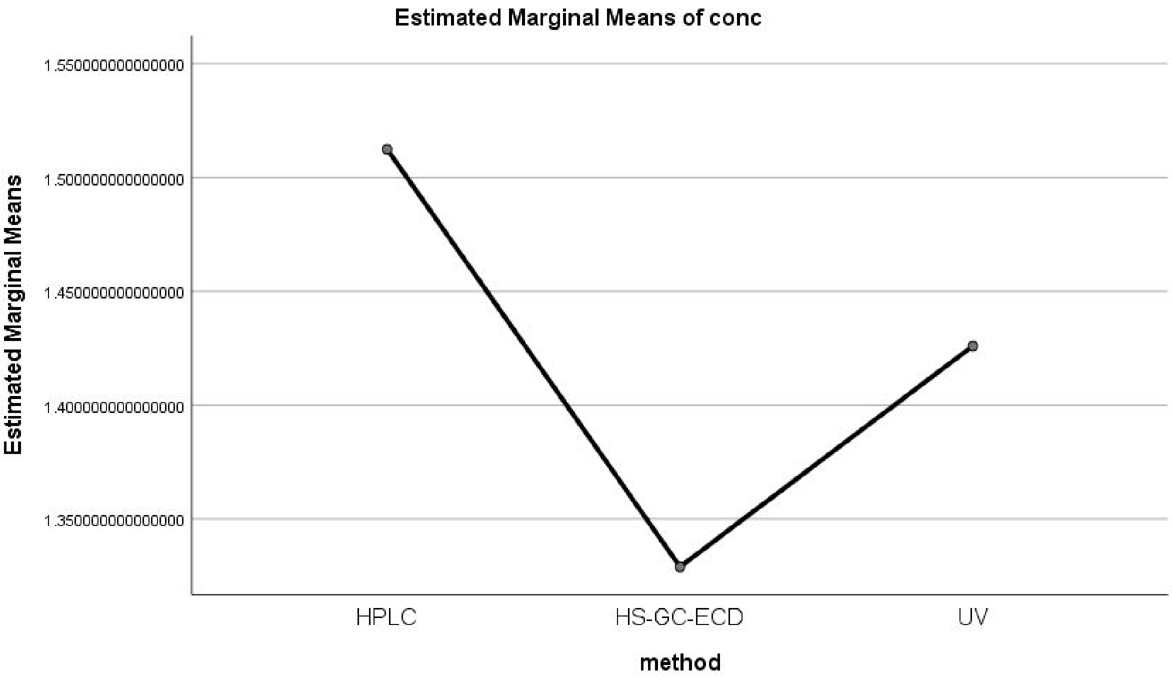

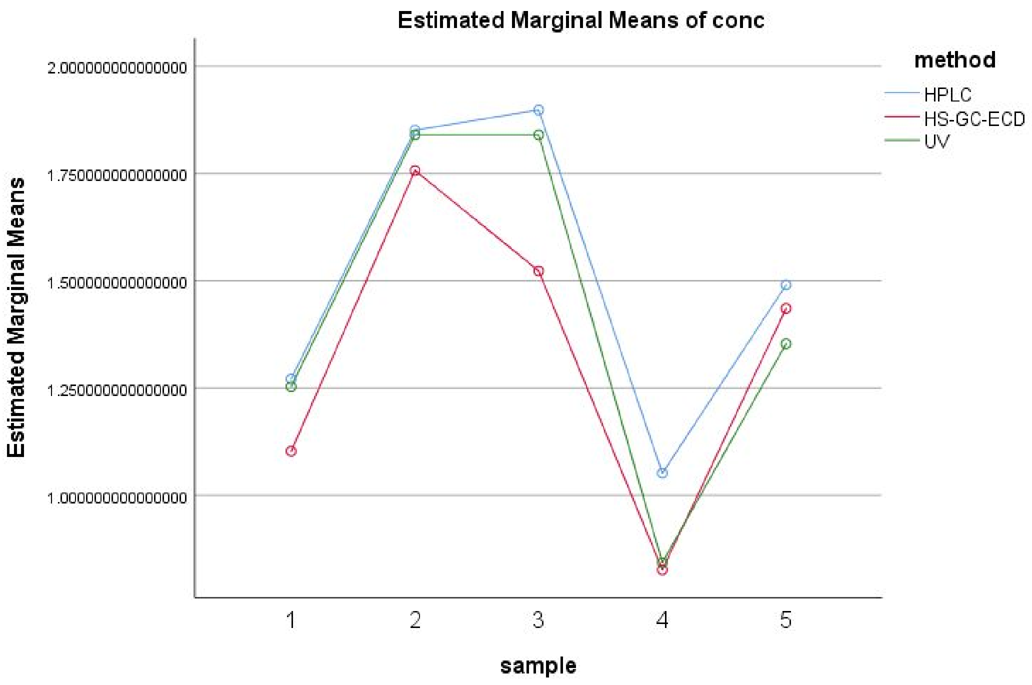
Estimated Marginal Means of conc
method
HPLC
HS-GC-ECD
UV
Estimated Marginal Means
2.000000000000000
1.750000000000000
1.500000000000000
1.250000000000000
1.000000000000000
1
2
3
4
5
sample

Univariate Analysis of Variance Acetaldehyde

Between-Subjects Factors

		N
sample	1	9
	2	9
	3	9
	4	9
	5	9
method	GC-MS	15
	HPLC	15
	HS-GC-ECD	15

Descriptive Statistics

Dependent Variable: conc

sample	method	Mean	Std. Deviation	N
1	GC-MS	0.082	0.005	3
	HPLC	0.153	0.037	3
	HS-GC-ECD	0.082	0.003	3
	Total	0.106	0.040	9
2	GC-MS	0.318	0.011	3
	HPLC	0.242	0.042	3
	HS-GC-ECD	0.299	0.013	3
	Total	0.286	0.041	9
3	GC-MS	0.017	0.001	3
	HPLC	0.059	0.010	3
	HS-GC-ECD	0.015	0.003	3

	Total	0.030	0.022	9
4	GC-MS	0.449	0.025	3
	HPLC	0.380	0.012	3
	HS-GC-ECD	0.444	0.006	3
	Total	0.424	0.036	9
5	GC-MS	0.588	0.093	3
	HPLC	0.526	0.044	3
	HS-GC-ECD	0.603	0.010	3
	Total	0.572	0.063	9
Total	GC-MS	0.291	0.226	15
	HPLC	0.272	0.173	15
	HS-GC-ECD	0.288	0.227	15
	Total	0.284	0.206	45

Levene's Test of Equality of Error Variances[a,b]

		Levene Statistic	df1	df2	Sig.
conc	Based on Mean	7.633	14	30	.000
	Based on Median	.847	14	30	.618
	Based on Median and with adjusted df	.847	14	4.252	.637
	Based on trimmed mean	6.518	14	30	.000

Tests the null hypothesis that the error variance of the dependent variable is equal across groups.

a. Dependent variable: conc

b. Design: Intercept + sample_A + method_A + sample_A * method_A

Tests of Between-Subjects Effects

Dependent Variable: conc

Source	Type III Sum of Squares	df	Mean Square	F	Sig.	Partial Eta Squared
Corrected Model	1.831[a]	14	.131	130.274	.000	.984
Intercept	3.622	1	3.622	3608.591	.000	.992
sample_A	1.789	4	.447	445.482	.000	.983
method_A	.003	2	.002	1.532	.232	.093
sample_A * method_A	.039	8	.005	4.856	.001	.564
Error	.030	30	.001			
Total	5.483	45				
Corrected Total	1.861	44				

a. R Squared = .984 (Adjusted R Squared = .976)

Estimated Marginal Means

1. Grand Mean

Dependent Variable: conc

Mean	Std. Error	95% Confidence Interval	
		Lower Bound	Upper Bound
.284	.005	.274	.293

2. sample

Estimates

Dependent Variable: conc

sample	Mean	Std. Error	95% Confidence Interval	
			Lower Bound	Upper Bound
1	.106	.011	.084	.127
2	.286	.011	.265	.308
3	.030	.011	.009	.052
4	.424	.011	.403	.446
5	.572	.011	.551	.594

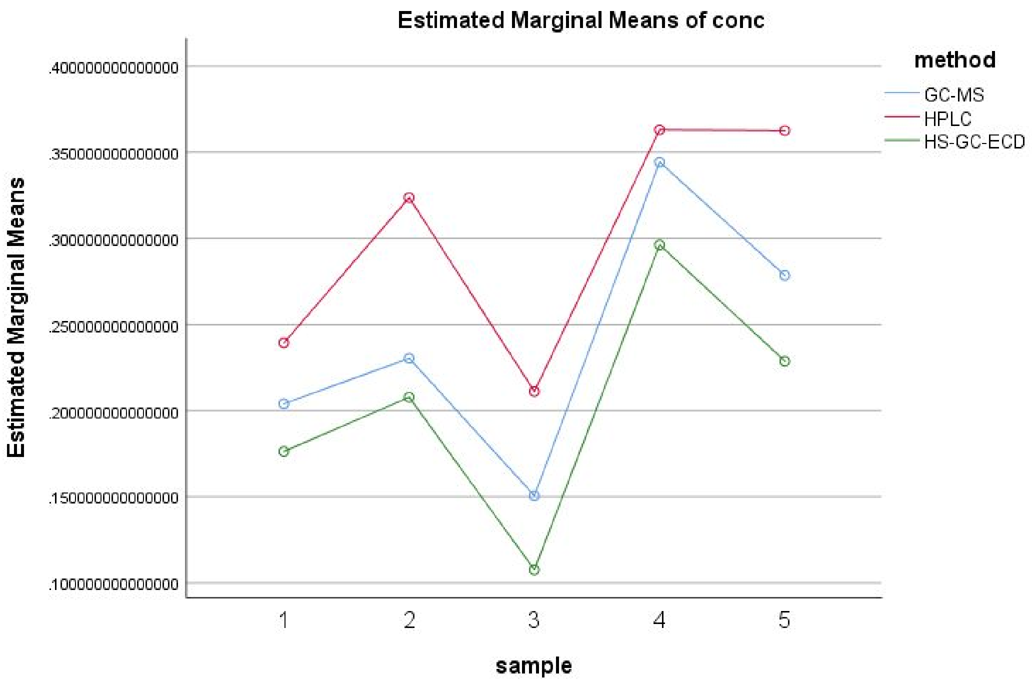
Estimated Marginal Means of conc
Estimated Marginal Means
.400000000000000
.350000000000000
.300000000000000
.250000000000000
.200000000000000
.150000000000000
.100000000000000
1
2
3
4
5
sample
method
GC-MS
HPLC
HS-GC-ECD

Univariate Analysis of Variance Furfural

Between-Subjects Factors

		N
sample	1	9
	2	9
	3	9
	4	9
	5	9
method	GC-MS	15
	HPLC	15
	HS-GC-ECD	15

Descriptive Statistics

Dependent Variable: conc

sample	method	Mean	Std. Deviation	N
1	GC-MS	0.204	0.003	3
	HPLC	0.239	0.014	3
	HS-GC-ECD	0.176	0.009	3
	Total	0.207	0.029	9
2	GC-MS	0.230	0.005	3
	HPLC	0.324	0.023	3
	HS-GC-ECD	0.208	0.012	3
	Total	0.254	0.055	9
3	GC-MS	0.151	0.008	3
	HPLC	0.211	0.028	3
	HS-GC-ECD	0.108	0.006	3
	Total	0.156	0.047	9
4	GC-MS	0.344	0.003	3
	HPLC	0.363	0.012	3
	HS-GC-ECD	0.296	0.027	3
	Total	0.335	0.033	9
5	GC-MS	0.278	0.001	3
	HPLC	0.363	0.004	3
	HS-GC-ECD	0.229	0.024	3
	Total	0.290	0.060	9
Total	GC-MS	0.242	0.068	15
	HPLC	0.300	0.067	15
	HS-GC-ECD	0.203	0.066	15
	Total	0.248	0.077	45

Levene's Test of Equality of Error Variances[a,b]

		Levene Statistic	df1	df2	Sig.
conc	Based on Mean	2.807	14	30	.009
	Based on Median	1.023	14	30	.458
	Based on Median and with adjusted df	1.023	14	12.529	.488
	Based on trimmed mean	2.656	14	30	.012

Tests the null hypothesis that the error variance of the dependent variable is equal across groups.

a. Dependent variable: conc

b. Design: Intercept + sample_B + method_B + sample_B * method_B

Tests of Between-Subjects Effects

Dependent Variable: conc

Source	Type III Sum of Squares	df	Mean Square	F	Sig.	Partial Eta Squared
Corrected Model	.254[a]	14	.018	80.746	.000	.974
Intercept	2.773	1	2.773	12340.535	.000	.998
sample_B	.175	4	.044	194.186	.000	.963
method_B	.071	2	.036	158.124	.000	.913
sample_B * method_B	.008	8	.001	4.681	.001	.555
Error	.007	30	.000			
Total	3.034	45				
Corrected Total	.261	44				

a. R Squared = .974 (Adjusted R Squared = .962)

Estimated Marginal Means

1. Grand Mean

Dependent Variable: conc

		95% Confidence Interval	
Mean	Std. Error	Lower Bound	Upper Bound
.248	.002	.244	.253

2. sample

Estimates

Dependent Variable: conc

			95% Confidence Interval	
sample	Mean	Std. Error	Lower Bound	Upper Bound
1	.207	.005	.196	.217
2	.254	.005	.244	.264
3	.156	.005	.146	.167
4	.335	.005	.324	.345
5	.290	.005	.280	.300

Pairwise Comparisons

Dependent Variable: conc

(I) sample	(J) sample	Mean Difference (I-J)	Std. Error	Sig.[b]	95% Confidence Interval for Difference[b]	
					Lower Bound	Upper Bound
1	2	-.047*	.007	.000	-.069	-.026
	3	.050*	.007	.000	.029	.072
	4	-.128*	.007	.000	-.149	-.107
	5	-.083*	.007	.000	-.105	-.062
2	1	.047*	.007	.000	.026	.069
	3	.098*	.007	.000	.076	.119
	4	-.081*	.007	.000	-.102	-.059
	5	-.036*	.007	.000	-.057	-.015
3	1	-.050*	.007	.000	-.072	-.029
	2	-.098*	.007	.000	-.119	-.076
	4	-.178*	.007	.000	-.200	-.157
	5	-.134*	.007	.000	-.155	-.112
4	1	.128*	.007	.000	.107	.149
	2	.081*	.007	.000	.059	.102
	3	.178*	.007	.000	.157	.200
	5	.045*	.007	.000	.023	.066
5	1	.083*	.007	.000	.062	.105
	2	.036*	.007	.000	.015	.057
	3	.134*	.007	.000	.112	.155
	4	-.045*	.007	.000	-.066	-.023

Based on estimated marginal means

*. The mean difference is significant at the .05 level.

b. Adjustment for multiple comparisons: Bonferroni.

Univariate Tests

Dependent Variable: conc

	Sum of Squares	df	Mean Square	F	Sig.	Partial Eta Squared
Contrast	.175	4	.044	194.186	.000	.963
Error	.007	30	.000			

The F tests the effect of sample. This test is based on the linearly independent pairwise comparisons among the estimated marginal means.

3. method

Estimates

Dependent Variable: conc

			95% Confidence Interval	
method	Mean	Std. Error	Lower Bound	Upper Bound
GC-MS	.242	.004	.234	.249
HPLC	.300	.004	.292	.308
HS-GC-ECD	.203	.004	.195	.211

Pairwise Comparisons

Dependent Variable: conc

(I) method	(J) method	Mean Difference (I-J)	Std. Error	Sig.[b]	95% Confidence Interval for Difference[b]	
					Lower Bound	Upper Bound
GC-MS	HPLC	-.058*	.005	.000	-.072	-.045
	HS-GC-ECD	.038*	.005	.000	.024	.052
HPLC	GC-MS	.058*	.005	.000	.045	.072
	HS-GC-ECD	.097*	.005	.000	.083	.111
HS-GC-ECD	GC-MS	-.038*	.005	.000	-.052	-.024
	HPLC	-.097*	.005	.000	-.111	-.083

Based on estimated marginal means

*. The mean difference is significant at the .05 level.

b. Adjustment for multiple comparisons: Bonferroni.

Univariate Tests

Dependent Variable: conc

	Sum of Squares	df	Mean Square	F	Sig.	Partial Eta Squared
Contrast	.071	2	.036	158.124	.000	.913
Error	.007	30	.000			

The F tests the effect of method. This test is based on the linearly independent pairwise comparisons among the estimated marginal means.

4. sample * method

Dependent Variable: conc

sample	method	Mean	Std. Error	95% Confidence Interval	
				Lower Bound	Upper Bound
1	GC-MS	.204	.009	.186	.222
	HPLC	.239	.009	.222	.257
	HS-GC-ECD	.176	.009	.159	.194
2	GC-MS	.230	.009	.213	.248
	HPLC	.324	.009	.306	.341
	HS-GC-ECD	.208	.009	.190	.225
3	GC-MS	.151	.009	.133	.168
	HPLC	.211	.009	.193	.229
	HS-GC-ECD	.108	.009	.090	.125
4	GC-MS	.344	.009	.327	.362
	HPLC	.363	.009	.345	.381
	HS-GC-ECD	.296	.009	.279	.314
5	GC-MS	.278	.009	.261	.296
	HPLC	.363	.009	.345	.380
	HS-GC-ECD	.229	.009	.211	.246

Post Hoc Tests method

Multiple Comparisons

Dependent Variable: conc

Tukey HSD

(I) method	(J) method	Mean Difference (I-J)	Std. Error	Sig.	95% Confidence Interval	
					Lower Bound	Upper Bound
GC-MS	HPLC	-.058430194151844*	.005474137901670	.000	-.071925429048409	-.044934959255280
	HS-GC-ECD	.038216255260016*	.005474137901670	.000	.024721020363452	.051711490156581
HPLC	GC-MS	.058430194151844*	.005474137901670	.000	.044934959255280	.071925429048409
	HS-GC-ECD	.096646449411861*	.005474137901670	.000	.083151214515296	.110141684308425
HS-GC-ECD	GC-MS	-.038216255260016*	.005474137901670	.000	-.051711490156581	-.024721020363452
	HPLC	-.096646449411861*	.005474137901670	.000	-.110141684308425	-.083151214515296

Based on observed means.

The error term is Mean Square(Error) = .000.

*. The mean difference is significant at the .05 level.

Homogeneous Subsets

conc

Tukey HSD[a,b]

method	N	Subset		
		1	2	3
HS-GC-ECD	15	.203305966292633		
GC-MS	15		.241522221552649	
HPLC	15			.299952415704494
Sig.		1.000	1.000	1.000

Means for groups in homogeneous subsets are displayed.

Based on observed means.

The error term is Mean Square(Error) = .000.

a. Uses Harmonic Mean Sample Size = 15.000.

b. Alpha = .05.

Profile Plots

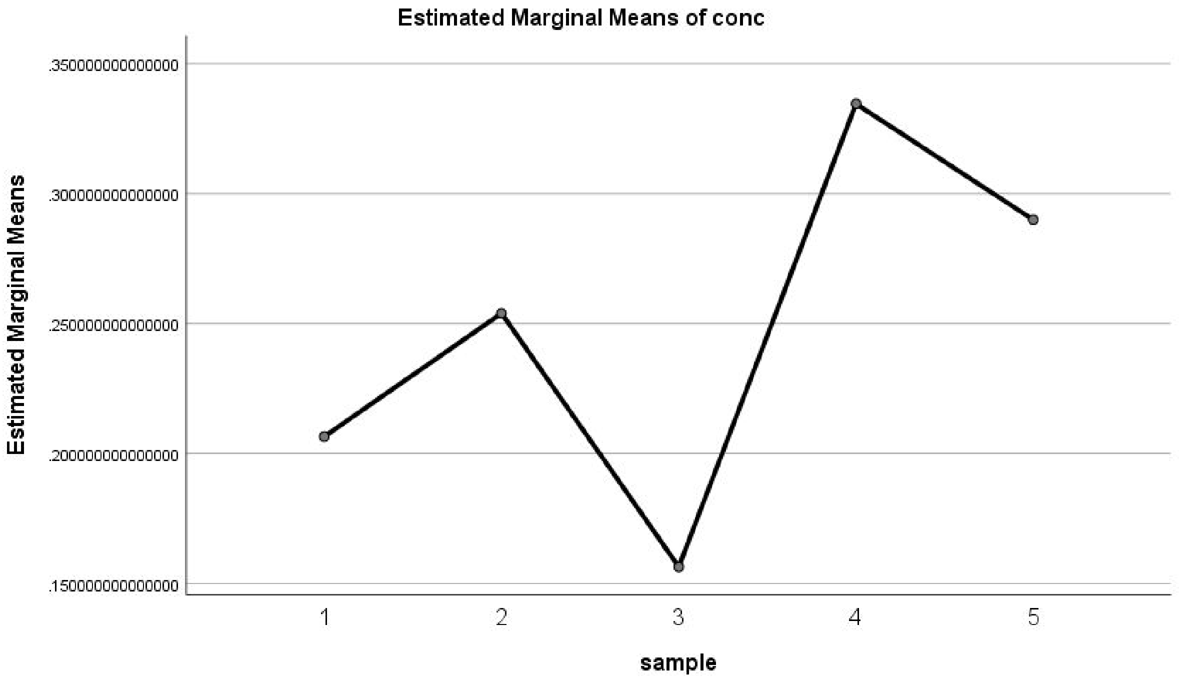

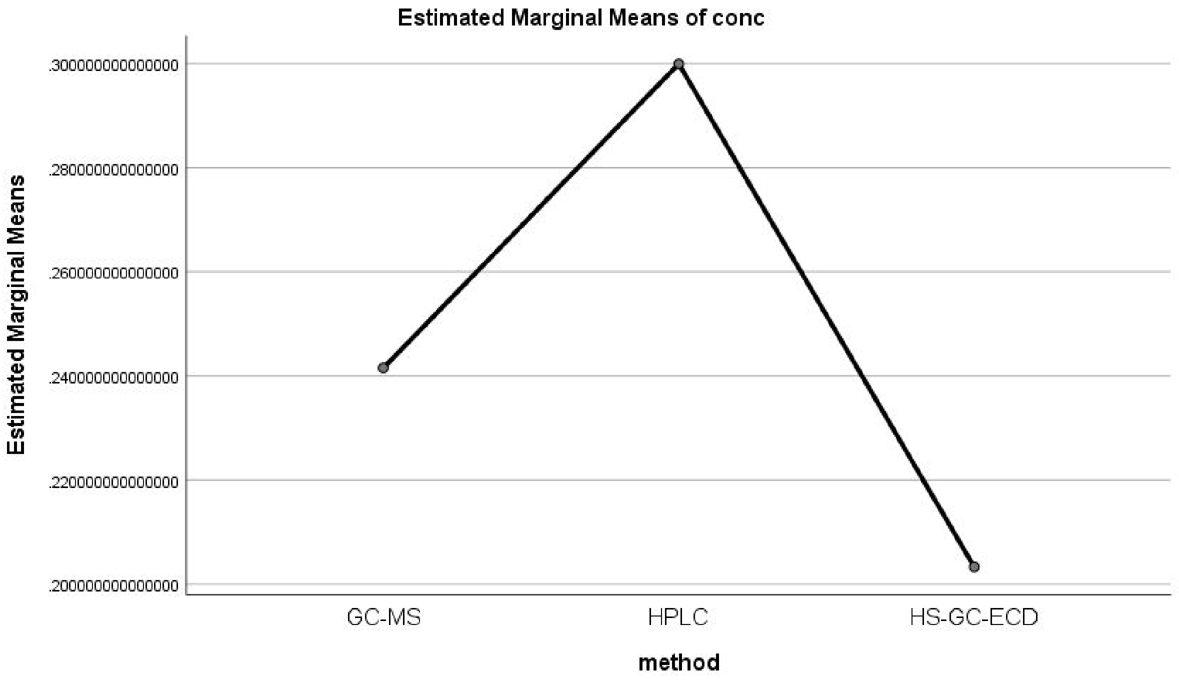

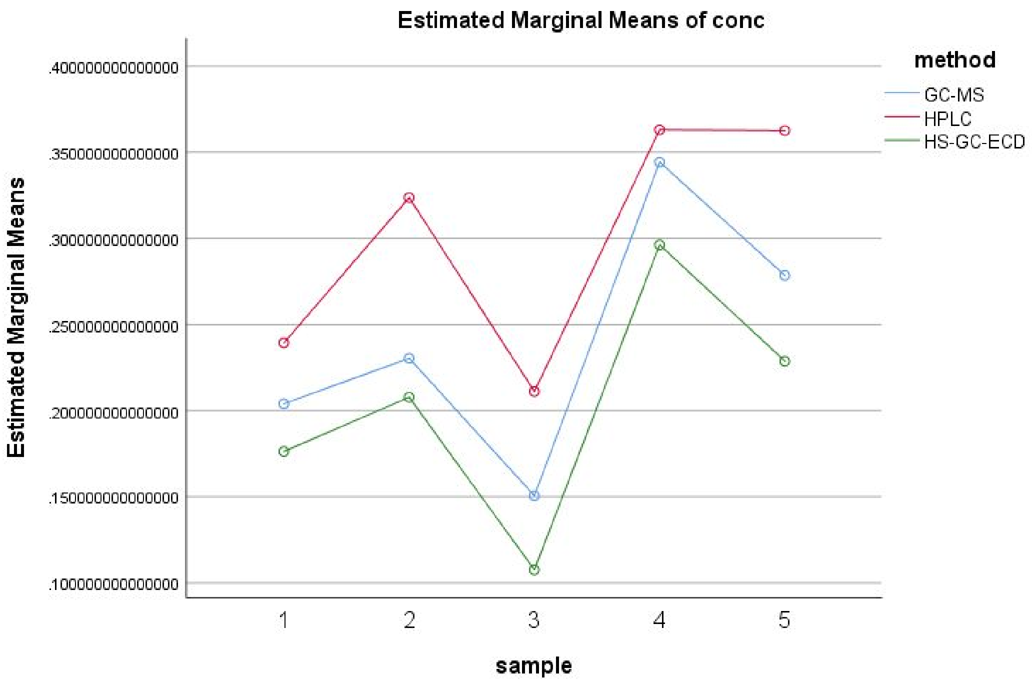
Estimated Marginal Means of conc
method
GC-MS
HPLC
HS-GC-ECD
Estimated Marginal Means
.400000000000000
.350000000000000
.300000000000000
.250000000000000
.200000000000000
.150000000000000
.100000000000000
1
2
3
4
5
sample
